# Serum neurofilament light chain levels are associated with depression among US adults: a cross-sectional analysis among US adults, 2013–2014

**DOI:** 10.1186/s12888-024-05964-0

**Published:** 2024-07-24

**Authors:** Ying Song, Huili Jiao, Qi Weng, Hang Liu, Li Yi

**Affiliations:** Department of Neurology, Peking University Shenzhen Hospital, Shenzhen, 518036 China

**Keywords:** Serum neurofilament light chain, Depression, NHANES, PHQ-9, Neurological disorders

## Abstract

**Background:**

Serum neurofilament light chain (sNfL) has been identified as a biomarker for neurologic diseases. However, sNfL remains unknown to be responsible for depression.

**Aims:**

The aim of this research was to explore the relationship between sNfL levels and depression in US adults.

**Methods:**

In this cross-sectional survey of the general population, we investigated representative data involving 10,175 participants from the 2013–2014 cycle of the National Health and Nutrition Examination Survey (NHANES). Depression was diagnosed using the Patient Health Questionnaire-9 (PHQ-9). The effect of related factors on depression was analyzed by conducting a univariate analysis. Stratified analysis was utilized to detect the stability and sensitivity of the relationship. After adjusting for race, education, marital status, smoking status, body mass index (BMI), sleep duration, income, and a history of hypertension, sedentary behavior and stroke, multivariable linear regression was performed to demonstrate the correlation between sNfL and depression.

**Results:**

A total of 1301 individuals between the ages of 20 and 75 were involved in this investigation, of which 108 (8.3%) were diagnosed with depression. A significant positive correlation between sNfL and depression among adults in the US was observed by conducting univariable analyses. After adjusting for confounding factors, the multivariate analyses indicated that elevated sNfL levels might play a pivotal role in the development of depression (odds ratio (OR) = 3.0; 95% confidence interval (CI): (1.5, 6.1), *P* = 0.002).

**Conclusion:**

These results indicated that sNfL is closely linked to depression in a nationally representative individual. However, further studies are needed to confirm the biological mechanism as well as the clinical implications of sNfL and depression.

**Supplementary Information:**

The online version contains supplementary material available at 10.1186/s12888-024-05964-0.

## Introduction

Depression, the most prevalent psychiatric symptom, is distinguished by a diminished mood, absence of vitality, sorrow, sleeplessness, and an inability to derive pleasure from life [1]. Around 20% of the American population experiences depression at some point in their lives, leading to poor outcomes in psychosocial and quality of life [2, 3]. In addition, there is a strong link between depression and suicide. In America, the leading cause of suicides is mood disorder, especially depression, which accounts for almost a third of all deaths worldwide [4, 5]. Currently, treatment approaches for depression include pharmacologic treatments and nonpharmacologic therapies, such as psychotherapy, pharmacologic treatments, cognitive behavior therapy (CBT) and electroconvulsive therapy (ECT) [6–8]. However, up to one-third of individuals do not exhibit a positive response to those therapies. Additionally, among those who did respond, only a third achieved remission [9, 10]. Furthermore, it is impossible to escape the adverse reactions, which involve sexual dysfunction, decreased sex drive, headaches, digestive issues, feelings of unease, and restlessness [11]. ECT not only requires general anesthesia and must take comorbidities into account, but also has not examined evidence for treatment-resistant people [1]. Unlike many other neurologic diseases, there are no established blood biomarkers that can be used to predict the progression of depression, which makes diagnosis challenging. Therefore, it is urgent for us to discover effective biomarkers for the diagnosis and treatment of depression.

Neurofilament light chain (NfL) is a scaffolding protein of the neuronal cytoskeleton, and its elevated level reflects neuroaxonal damage [12]. As a marker of neurodegeneration, serum neurofilament light chain (sNfL) is released not only to cerebrospinal fluid (CSF) but also to blood [13, 14]. Thus, NfL levels in the blood are always be measured in the blood as well as CSF [15]. However, neurofilament levels in the blood always be measured by precise assay technology given that lumbar puncture is an invasive procedure [16]. Moreover, sNfL is well-established as a biomarker for disease prognosis and monitoring recurrences, including multiple sclerosis (MS), cognition decline, stroke, traumatic brain injury, Guillain-Barré syndrome (GBS) and primary psychiatric disorders (PPD) [17–21]. Therefore, the detection of NfL level in serum is widely utilized for evaluating neuroinflammatory and degenerative diseases.

However, based on our current understanding, there is no evidence indicating a connection between sNfL and depression within the entire population of the United States. It is therefore worthwhile to explore whether sNfL concentrations are a dependent predictor of depression progression. In this study, we presented the first known evidence for a correlation between sNfL and depression among the overall population of the United States from the 2013–2014 cycle of the NHANES database.

## Materials and methods

### Design and methods of the study

The NHANES program was created to investigate the health and nutritional status of the United States population by conducting a series of interviews, examinations and laboratory measurements, whose findings hold significant implications for discovering disease risk factors as well as effective interventions [22, 23].

**Fig. 1:**
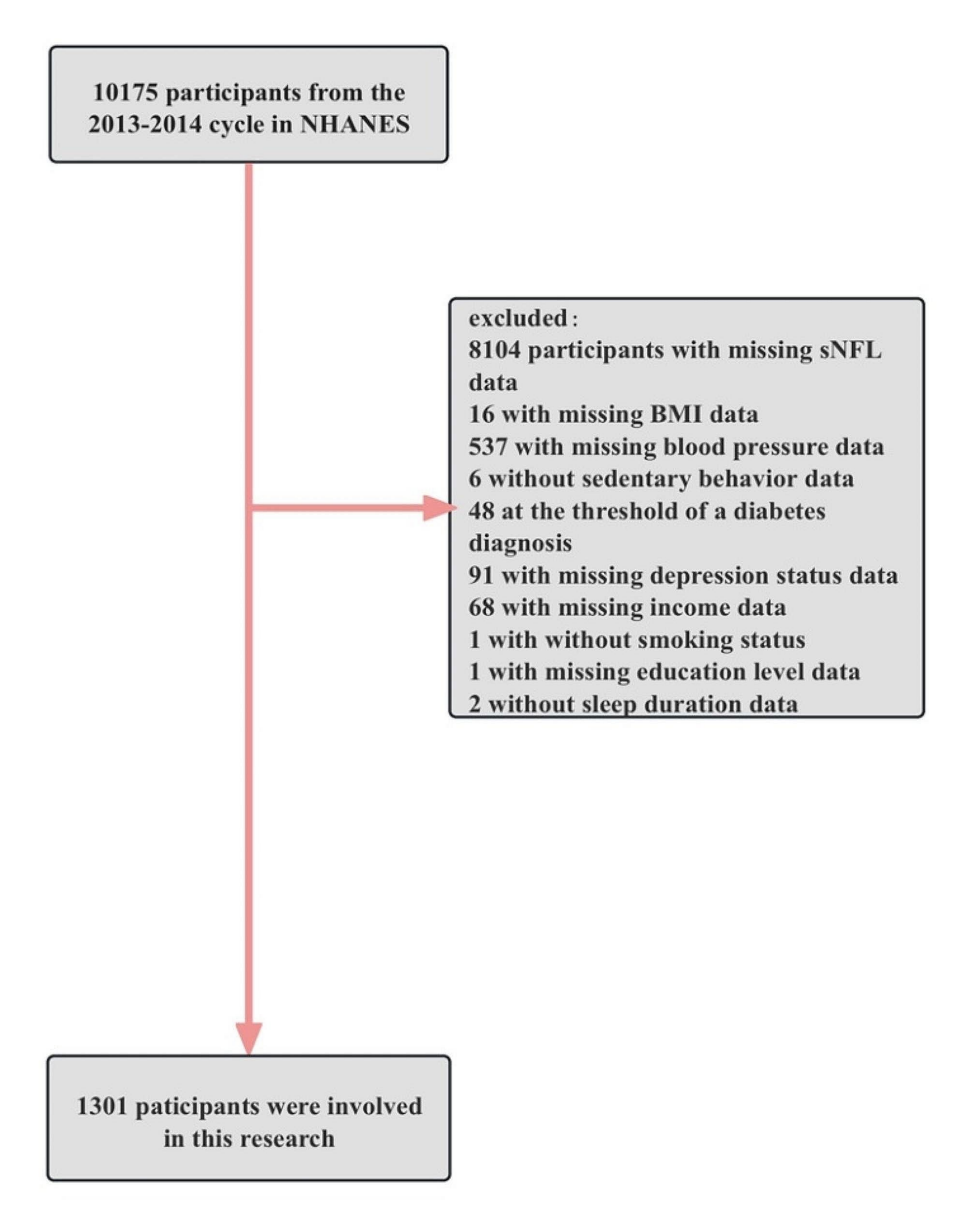
The workflow of the research. Abbreviations NHANES: National Health and Nutrition Examination Survey; BMI: body mass index; sNFL: serum neurofilament light chain

For this investigation, the 2013–2014 continuous cycle of the NHANES dataset was obtained. After excluding 8104 participants who had missing sNfL data, 16 participants with missing BMI data, 537 participants with missing blood pressure data, 6 participants without sedentary behavior data, 48 participants at the threshold of a diabetes diagnosis, 91 participants with missing depression status data, 68 participants with missing income data, 1 participant without smoking status and education levels data, and 2 participants without sleep duration data, a total of 1301 participants were eligible for this study. Figure 1 illustrates the sample selection flowchart.

### Serum neurofilament light chain measurement

This study collected serum samples from individuals aged 20–75 years in the NHANES 2013–2014 cycle, who gave informed consent for the utilization of the remaining specimens in subsequent research. Initially, the specimens are cultured with acridinium-ester (AE)-labeled NfL antibody, followed by the introduction of paramagnetic particles (PMP) coated with capture antibody into the sample. Subsequently, the formation of antigen-AE-labeled antibodies and PMP complexes were acquired. Additionally, the sample is supplemented with paramagnetic particles (PMP) that have a coating of capture antibody to obtain paramagnetic particles. After the unbound AE-labeled antibodies were isolated and eliminated, the chemiluminescence was triggered and the emission of light was measured.

### Assessment of depression symptoms

As a convenient tool, PHQ-9 was utilized to evaluate the frequency of depressive mood and anhedonia over a period of two weeks objectively [1, 24, 25]. The PHQ-9 assigned a score of 0 to 3 for each item (0 = none; 1 = some days; 2 = most days; 3 = almost every day), and the total scores for the PHQ-9 varied from 0 to 27 [26, 27]. In this study, participants were defined as having clinically significant depressive symptoms at a cutoff of ≥ 10, with a sensitivity and specificity of 74% and 91%, respectively [2, 25, 28].

### Other covariates

The current research examined the age (20–34 years, 35–60 years, 61–75 years), gender, race/ethnicity (Mexican American, other Hispanic, non-Hispanic white, non-Hispanic black, and other races), body mass index (normal weight:<25.0, overweight: [25.0, 30.0), obesity:≥30.0 kg/m^2^), educational levels (≤ 9th grade, 9-11th grade/includes 12th grade with no diploma, high school graduate/GED or equivalent, some college or AA degree, and college graduate or above), marital status (married, living with partner, widowed, divorced, separated, and never married), family poverty income ratio (< 1, [1,3), ≥ 3), sleep duration (< 7, [7,9), ≥ 9 h), hypertension (systolic blood pressure ≥ 130 mmHg and/or diastolic blood pressure ≥ 80 mmHg), smoking status, congestive heart failure, sedentary behavior, coronary heart disease and stroke [29–33].

### Statistical analysis

This study followed the Strengthening the Reporting of Observational Studies in Epidemiology (STROBE) guidelines. R software (4.1.3, http://www.Rproject.org) and EmpowerStats (version 2.0, http://www.empowerstats.com) were utilized for all the analyses. An overview of the study population was statistically described by sNfL concentrations (Table 1). Participants’ characteristics based on quartiles of sNfL levels were compared through Rao–Scott χ2 tests. SNfL levels were logarithmically transformed to achieve a normal distribution. Then, univariate and multivariate analyses were performed to analyze the relationship between sNfL levels and depression, as well as other outcomes. The multivariate linear regression was built after taking those confounders into consideration: race, education, marital status, BMI, blood pressure, sedentary behavior, sleep duration and having a history of diabetes. *P* < 0.05 was considered statistically significant. We also conducted a smooth curve fitting and a threshold effect analysis for enhancing the correlation between sNfL and PHQ-9 scores, after taking all confounders into consideration. For the threshold effects analysis, a log-likehood ratio test of less than 0.05 was considered the criterion for the presence of a non-linear relationship. Model I was conducted using liner regression, and Model II was performed using a two-piecewise linear regression (Additional file 1 and 2).

**Table 1:** Characteristics of participants by quartiles of sNfL

sNfL quartile					
	Q1	Q2	Q3	Q4	*P*-value
**Gender**					0.012
Male	137 (42.2%)	167 (52.7%)	172 (52.0%)	175 (53.4%)	
Female	188 (57.8%)	150 (47.3%)	159 (48.0%)	153 (46.6%)	
**Age(years)**					< 0.001
20–34	172 (52.9%)	89 (28.1%)	60 (18.1%)	27 (8.2%)	
35–60	148 (45.5%)	183 (57.7%)	171 (51.7%)	141 (43.0%)	
61–75	5 (1.5%)	45 (14.2%)	100 (30.2%)	160 (48.8%)	
**Race**					< 0.001
Mexican American	62 (19.1%)	45 (14.2%)	36 (10.9%)	37 (11.3%)	
Other Hispanic	29 (8.9%)	29 (9.1%)	38 (11.5%)	25 (7.6%)	
Non-Hispanic white	123 (37.8%)	133 (42.0%)	168 (50.8%)	171 (52.1%)	
Non-Hispanic black	63 (19.4%)	72 (22.7%)	35 (10.6%)	56 (17.1%)	
Non-Hispanic Asian	38 (11.7%)	33 (10.4%)	45 (13.6%)	31 (9.5%)	
Other Race (Including Multi-Racial)	10 (3.1%)	5 (1.6%)	9 (2.7%)	8 (2.4%)	
**Education**					0.188
Less than 9th grade	15 (4.6%)	21 (6.6%)	18 (5.4%)	23 (7.0%)	
9-11th grade	53 (16.3%)	42 (13.2%)	41 (12.4%)	41 (12.5%)	
High school graduate	69 (21.2%)	68 (21.5%)	61 (18.4%)	78 (23.8%)	
Some college or AA degree	100 (30.8%)	92 (29.0%)	102 (30.8%)	114 (34.8%)	
College graduate or above	88 (27.1%)	94 (29.7%)	109 (32.9%)	72 (22.0%)	
**Marital status**					< 0.001
Married	167 (51.4%)	162 (51.1%)	189 (57.1%)	189 (57.6%)	
Widowed	1 (0.3%)	5 (1.6%)	22 (6.6%)	28 (8.5%)	
Divorced	24 (7.4%)	36 (11.4%)	43 (13.0%)	48 (14.6%)	
Separated	7 (2.2%)	12 (3.8%)	8 (2.4%)	7 (2.1%)	
Never married	92 (28.3%)	74 (23.3%)	55 (16.6%)	34 (10.4%)	
Living with partner	34 (10.5%)	28 (8.8%)	14 (4.2%)	22 (6.7%)	
**BMI (kg/m** ^**2**^ **)**					0.026
Normal	98 (30.2%)	116 (36.6%)	111 (33.5%)	86 (26.2%)	
Overweight	96 (29.5%)	104 (32.8%)	114 (34.4%)	120 (36.6%)	
Obesity	131 (40.3%)	97 (30.6%)	106 (32.0%)	122 (37.2%)	
**Hypertension**					< 0.001
No	243 (74.8%)	214 (67.5%)	205 (61.9%)	173 (52.7%)	
Yes	82 (25.2%)	103 (32.5%)	126 (38.1%)	155 (47.3%)	
**Sedentary behavior(hours)**					0.539
No	187 (57.5%)	172 (54.3%)	180 (54.4%)	170 (51.8%)	
Yes	138 (42.5%)	145 (45.7%)	151 (45.6%)	158 (48.2%)	
**Diabetes**					< 0.001
No	315 (96.9%)	299 (94.3%)	287 (86.7%)	256 (78.0%)	
Yes	10 (3.1%)	18 (5.7%)	44 (13.3%)	72 (22.0%)	
**Depression**					0.012
No	306 (94.2%)	295 (93.1%)	305 (92.1%)	287 (87.5%)	
Yes	19 (5.8%)	22 (6.9%)	26 (7.9%)	41 (12.5%)	
**Congestive heart failure**					< 0.001
No	324 (99.7%)	313 (98.7%)	320 (96.7%)	311 (94.8%)	
Yes	1 (0.3%)	4 (1.3%)	11 (3.3%)	17 (5.2%)	
**Coronary heart disease**					< 0.001
No	325 (100.0%)	316 (99.7%)	319 (96.4%)	304 (92.7%)	
Yes	0 (0.0%)	1 (0.3%)	12 (3.6%)	24 (7.3%)	
**Stroke**					0.002
No	324 (99.7%)	312 (98.4%)	320 (96.7%)	313 (95.4%)	
Yes	1 (0.3%)	5 (1.6%)	11 (3.3%)	15 (4.6%)	
**Income**					0.665
Low income	126 (38.8%)	107 (33.8%)	111 (33.5%)	111 (33.8%)	
Median income	50 (15.4%)	45 (14.2%)	51 (15.4%)	45 (13.7%)	
High income	149 (45.8%)	165 (52.1%)	169 (51.1%)	172 (52.4%)	
**Smoking status**					0.005
No	204 (62.8%)	179 (56.5%)	174 (52.6%)	163 (49.7%)	
Yes	121 (37.2%)	138 (43.5%)	157 (47.4%)	165 (50.3%)	
**Sleep duration(hours)**					0.382
< 7	116 (35.7%)	132 (41.6%)	123 (37.2%)	119 (36.3%)	
[7,9)	187 (57.5%)	170 (53.6%)	180 (54.4%)	182 (55.5%)	
>=9	22 (6.8%)	15 (4.7%)	28 (8.5%)	27 (8.2%)	

Abbreviations sNFL: serum neurofilament light chain; BMI: body mass index. Reported are results of Rao–Scott χ2 test. Interpretation of *p* values: *p* < 0.05

## Results

### The baseline characteristics of study population

Among the 10175 participants in the 2013–2014 NHANES, 1301 were involved (Fig. 1). The descriptive characteristics of the participants are displayed in Table 1, categorized by sNfL quartiles. The participants were divided into four groups based on their sNfL levels: Q1 (sNfL = 2.8–8.3 pg/mL), Q2 (sNfL = 8.4–12.3 pg/mL), Q3 (sNfL = 12.4–18.8 pg/mL) and Q4 (sNfL > 18.8 pg/mL). Participants ranged in age from 20 to 75, and 50% (*n* = 650) are male. Around 8.3% (*n* = 108) exhibited signs of depression (PHQ-9 score > = 10), while the remaining 91.7% (*n* = 1193) did not show any depressive symptoms (PHQ-9 score < 10). Interesting, there was a strong positive association between age and sNfL levels, which coincides with previous theories. Individuals with elevated sNfL levels were more likely to be male, older, Hispanic, married, obese and there was a significantly lower proportion of participants who had never smoked. Furthermore, participants exhibiting elevated sNfL levels demonstrated a higher occurrence of comorbidities, such as high blood pressure, sedentary behavior, diabetes, stroke, congestive heart failure and coronary heart disease, compared with lower sNfL levels.

### Association of sNfL and the risk of depression

To examine the correlation between sNfL and depression, a univariate analysis was conducted (Table 2). The results showed that age (35–60 years: OR = 2.2, 95% CI: (1.2, 3.9), *P* = 0.010; 61–75 years: OR = 2.9 95% CI: (1.6, 5.4), *P* < 0.001), gender (female: OR = 1.8, 95% CI: (1.2, 2.7), *P* = 0.005), income (OR = 0.5, 95% CI: (0.3, 0.9) *P* = 0.03), smoking status (OR = 1.5, 95% CI: (1.0, 2.3), *P* = 0.030), had a disease history of stroke (OR = 2.6, 95% CI: (1.1, 6.6), P 0.037), congestive heart failure (OR = 4.4, 95% CI: (2.0, 9.8), *P* < 0.001), coronary heart disease (OR = 5.1, 95% CI: (2.4, 10.6), *P* < 0.001) and log-transformed serum NFL concentrations (OR = 3.4, 95% CI: (1.8, 6.4), *P* < 0.001) had a statistical difference with high prevalence of depression. Stratified analyses (Table 3) were conducted for age, gender, race, education level, marital status, income, BMI, smoking status, blood pressure, sedentary behavior, sleep duration and complications such as congestive heart failure, coronary heart disease, stroke and diabetes. It turned out that despite the OR values fluctuated among subgroups of the population, there was a good deal of consistency in our analysis (OR > 1), suggesting that the results were stable and sensitive. Additionally, it was relatively stable in some stratifications, especially in age, smoking status and sedentary behavior, although high heterogeneity was observed in terms of race, education, income, marital status, sleep duration, and relevant disease histories of diabetes, congestive heart failure, coronary heart disease and stroke.

**Table 2:** Univariate analysis of log-transformed sNfL and depression among American adults

	Statistics	OR (95% CI)	*P*-value
Gender			
Male	651 (50.0%)	1.0	
Female	650 (50.0%)	1.8 (1.2, 2.7)	0.005
**Age(years)**			
20–34	348 (26.7%)	1.0	
35–60	643 (49.4%)	2.2 (1.2, 3.9)	0.010
61–75	310 (23.8%)	2.9 (1.6, 5.4)	< 0.001
**Race**			
Mexican American	180 (13.8%)	1.0	
Other Hispanic	121 (9.3%)	1.2 (0.5, 2.7)	0.686
Non-Hispanic white	595 (45.7%)	1.3 (0.7, 2.3)	0.464
Non-Hispanic black	226 (17.4%)	1.0 (0.5, 2.1)	0.945
Non-Hispanic Asian	147 (11.3%)	0.2 (0.1, 0.9)	0.031
Other Race (Including Multi-Racial)	32 (2.5%)	2.2 (0.7, 6.6)	0.161
**Education**			
Less than 9th grade	77 (5.9%)	1.0	
9-11th grade	177 (13.6%)	0.9 (0.4, 2.1)	0.824
High school graduate	276 (21.2%)	0.8 (0.4, 1.8)	0.626
Some college or AA degree	408 (31.4%)	0.8 (0.4, 1.8)	0.615
College graduate or above	363 (27.9%)	0.3 (0.1, 0.7)	0.005
**Marital status**			
Married	707 (54.3%)	1.0	
Widowed	56 (4.3%)	2.1 (1.0, 4.8)	0.062
Divorced	151 (11.6%)	2.3 (1.4, 3.9)	0.002
Separated	34 (2.6%)	1.7 (0.6, 5.1)	0.328
Never married	255 (19.6%)	0.9 (0.5, 1.5)	0.614
Living with partner	98 (7.5%)	0.8 (0.4, 2.0)	0.693
**BMI (kg/m** ^**2**^ **)**			
Normal	411 (31.6%)	1.0	
Overweight	434 (33.4%)	1.5 (0.8, 2.7)	0.167
Obesity	456 (35.0%)	2.8 (1.6, 4.7)	< 0.001
**Hypertension**			
No	835 (64.2%)	1.0	
Yes	466 (35.8%)	0.9 (0.6, 1.4)	0.724
**Sedentary behavior(hours)**			
No	709 (54.5%)	1.0	
Yes	592 (45.5%)	1.1 (0.7, 1.6)	0.708
**Diabetes**			
No	1157 (88.9%)	1.0	
Yes	144 (11.1%)	1.6 (0.9, 2.7)	0.109
**Congestive heart failure**			
No	1268 (97.5%)	1.0	
Yes	33 (2.5%)	4.4 (2.0, 9.8)	< 0.001
**Coronary heart disease**			
No	1264 (97.2%)	1.0	
Yes	37 (2.8%)	5.1 (2.4, 10.6)	< 0.001
**Stroke**			
No	1269 (97.5%)	1.0	
Yes	32 (2.5%)	2.6 (1.1, 6.6)	0.037
**Income**			
Low income	455 (35.0%)	1.0	
Median income	191 (14.7%)	0.5 (0.3, 0.9)	0.030
High income	655 (50.3%)	0.3 (0.2, 0.5)	< 0.001
**Smoking status**			
No	720 (55.3%)	1.0	
Yes	581 (44.7%)	1.5 (1.0, 2.3)	0.030
**Sleep duration(hours)**			
<7	490 (37.7%)	1.0	
[7,9)	719 (55.3%)	0.6 (0.4, 1.0)	0.028
>=9	92 (7.1%)	1.1 (0.5, 2.2)	0.847
**sNfL(pg/mL)**	1.1 ± 0.3	3.4 (1.8, 6.4)	< 0.001

Abbreviations sNFL: serum neurofilament light chain; BMI: body mass index; OR: odds ratio; 95% CI:95% confidence interval. Interpretation of p values: *p* < 0.05 was considered significant

**Table 3:** Associations between log-transformed sNfL and depression by stratified analysis

Subgroup	*N*	OR (95% CI)	*P*-value
Gender			
Male	651	5.4 (1.9, 15.1)	0.001
Female	650	2.8 (1.3, 6.3)	0.012
**Age(years)**			
20–34	348	1.5 (0.2, 12.9)	0.691
35–60	643	2.4 (0.9, 6.0)	0.066
61–75	310	3.2 (0.8, 12.5)	0.087
**Race**			
Mexican American	180	1.6 (0.3, 9.8)	0.619
Other Hispanic	121	4.0 (0.4, 39.5)	0.239
Non-Hispanic white	595	5.2 (2.1, 12.6)	< 0.001
Non-Hispanic black	226	1.8 (0.4, 8.4)	0.478
Non-Hispanic Asian	147	2.2 (0.0, 191.6)	0.736
Other Race (Including Multi-Racial)	32	1.0 (0.1, 17.9)	0.984
**Education**			
Less than 9th grade	77	3.0 (0.3, 32.9)	0.359
9-11th grade	177	1.0 (0.2, 6.2)	0.996
High school graduate	276	5.0 (1.4, 17.3)	0.012
Some college or AA degree	408	3.4 (1.3, 8.8)	0.013
College graduate or above	363	3.8 (0.5, 27.3)	0.184
**Marital status**			
Married	707	5.6 (2.3, 13.7)	< 0.001
Widowed	56	10.2 (0.4, 254.0)	0.157
Divorced	151	1.2 (0.3, 5.7)	0.784
Separated	34	1.0 (0.0, 107.6)	0.990
Never married	255	0.9 (0.1, 6.0)	0.942
Living with partner	98	1.7 (0.1, 34.8)	0.735
**BMI (kg/m** ^**2**^ **)**			
Normal	411	7.5 (1.9, 30.0)	0.004
Overweight	434	4.0 (1.0, 15.6)	0.045
Obesity	456	2.3 (1.0, 5.1)	0.053
**Hypertension**			
No	835	4.1 (1.8, 9.3)	< 0.001
Yes	466	2.8 (1.0, 8.1)	0.062
**Sedentary behavior(hours)**			
No	709	3.7 (1.6, 8.7)	0.002
Yes	592	2.9 (1.1, 7.7)	0.029
**Diabetes**			
No	1157	3.7 (1.8, 7.7)	< 0.001
Yes	144	1.5 (0.3, 7.8)	0.633
**Congestive heart failure**			
No	1268	3.2 (1.6, 6.2)	< 0.001
Yes	33	0.7 (0.0, 15.6)	0.834
**Coronary heart disease**			
No	1264	2.9 (1.5, 5.6)	0.002
Yes	37	2.3 (0.1, 69.6)	0.641
**Stroke**			
No	1269	3.0 (1.5, 5.8)	0.001
Yes	32	8.3 (0.4, 155.6)	0.157
**Income**			
Low income	455	2.3 (1.0, 5.4)	0.053
Median income	191	4.4 (0.7, 28.2)	0.116
High income	655	7.1 (2.3, 21.7)	< 0.001
**Smoking status**			
No	720	2.7 (1.0, 6.8)	0.041
Yes	581	3.8 (1.6, 9.3)	0.003
**Sleep duration(hours)**			
<7	490	2.0 (0.7, 5.6)	0.201
[7,9)	719	5.7 (2.3, 14.4)1	< 0.00
>=9	92	2.3 (0.4, 13.5)	0.355

Abbreviations sNFL: serum neurofilament light chain; BMI: body mass index; OR: odds ratio; 95% CI:95% confidence interval. Interpretation of p values: *p* < 0.05 was considered significant

### Multivariable-Adjusted Associations Between sNfL and depression

To evaluate the independent effects of log-transformed sNfL on depression, we conducted a multivariable analysis. Table 4 shows the significant predictors of depression included age (35–60 years: OR = 2.0, 95% CI: (1.0, 3.8), *P* = 0.038; 61–75 years: OR = 2.7, 95% CI: (1.3, 5.7), *P* = 0.010), sex (female: OR = 1.6, 95% CI: (1.0, 2.4), *P* = 0.042), income (median income: OR = 0.5, 95% CI: (0.3, 0.9), *P* = 0.032; high income: OR = 0.4, 95% CI: (0.2, 0.6), *P* < 0.001), congestive heart failure (OR = 3.2, 95% CI: (1.4, 7.6), *P* = 0.007), coronary heart disease (OR = 5.1, 95% CI: (2.3, 11.6), *P* < 0.001), and log-transformed sNfL levels (OR = 3.0, 95% CI: (1.5, 6.1), *P* = 0.002), and the result was stable after adjusted for race, marital status, education, BMI, sedentary behaviors, diabetes, hypertension, sleep duration. However, there was no significant difference in smoking status (OR = 1.3, 95% CI: (0.9, 2.0), *P* = 0.209) and stroke (OR = 1.9, 95% CI: (0.7, 5.0), *P* = 0.201).

**Table 4:** Multivariate logistic model of the predictors of depression

Exposure	OR (95% CI)	*P*-value
**Gender**		
Male	1.0	
Female	1.6 (1.0, 2.4)	**0.042**
**Age (years)**		
20–34	1.0	
35–60	2.0 (1.0, 3.8)	**0.038**
61–75	2.7 (1.3, 5.7)	**0.010**
**sNfL(pg/mL)**	3.0 (1.5, 6.1)	**0.002**
**Congestive heart failure**		
No	1.0	
Yes	3.2 (1.4, 7.6)	**0.007**
**Coronary heart disease**		
No	1.0	
Yes	5.1 (2.3, 11.6)	**< 0.001**
**Stroke**		
No	1.0	
Yes	1.9 (0.7, 5.0)	0.201
**Income**		
Low income	1.0	
Midian income	0.5 (0.3, 0.9)	**0.032**
High income	0.4 (0.2, 0.6)	< **0.001**
**Smoking status**		
No	1.0	
Yes	1.3 (0.9, 2.0)	0.209

Abbreviations sNFL: serum neurofilament light chain; BMI: body mass index; OR: odds ratio; 95% CI:95% confidence interval; OR for depression and 95% CI for a unit increase of log-transformed sNfL were adjusted for race, marital status, education, BMI, sedentary behaviors, diabetes, hypertension, sleep duration. Interpretation of p values: *p* < 0.05 was considered significant

Based on the results of regression analysis, a smooth curve fitting and a curve fitting analysis were performed to explore the non-liner relationship between the log10 transformation sNfL and PHQ-9 scores (Additional file 1 and 2). A. Figure 1 displayed a non-liner relationship between log-transformed serum NfL levels (pg/mL) and PHQ-9 scores (log-likelihood ratio < 0.001). A. Table 1 showed the threshold effect analysis of the log-transformed sNfL (pg/mL) on PHQ-9 scores. The results demonstrated that the turning point of log-transformed serum NfL levels was 1.5 pg/mL. When the log-transformed serum NfL levels less than 1.5 pg/mL, the effect value was 0.3 (95% CI = -2.0-2.5, *P* = 0.807). When the log-transformed serum NfL levels surpassed 1.5 pg/mL, the effect value was 8.9 (95% CI = 3.1–14.7, *P* = 0.003). The result showed the *P*-value of log-likehood ratio test (LR Test) < 0.001, indicating Model II is significant different from Model I. Moreover, from the overall smoothing curve, the relationship was still significant (β = 1.9, 95% CI = 0.1–3.8, *P* = 0.039).

## Discussion

As the most abundant and soluble subunit of the class IV intermediate filament protein family (including neurofilament heavy protein, neurofilament medium protein, neurofilament light protein and alpha-internexin), NFL levels are responsible for the stability of the neuronal structure [19, 34, 35]. SNfL, the core of mature neurofilament, is released into not only CSF but also blood when it comes to neuroaxonal damage [36]. However, considering cerebrospinal fluid collection by lumbar puncture is an invasive and complex procedure, measures of NfL can be obtained from blood NfL with higher accuracy and safety. SNfL may emerged as a diagnostic and prognostic biomarker for neuronal damage, whose levels are positively related to recurrence or new magnetic resonance imaging (MRI) lesions and negatively related to effective treatment [14]. However, sNfL has never been explored as a potential therapeutic response, diagnostic, prognostic or monitoring biomarker in depression.

This is the first epidemiological study to examine the relationships between sNfL and depression among adult samples from the US general population. Firstly, we explored whether participants with high sNfL levels (*n* = 41, 12.5%) had significant greater risk of depression characterized compared with those with low sNfL levels (*n* = 19, 5.8%) (Table 1). In addition, based on the results, older people are more likely to suffer from depression, which is consistent with previous theories that sNfL concentration increases with age. Then, univariate analysis showed that log-transformed sNfL levels differed significantly in subjects with and without depression (OR = 3.4, 95% CI: (1.8, 6.4), *P* < 0.001; Table 2), which demonstrated that log-transformed serum NfL levels are positive-related with depression. Moreover, the relationship is stable after stratified analysis was conducted (Table 3). Besides, multivariate analysis showed that log-transformed sNfL levels can be an independent factor for the prevalence of depression after taking race, marital status, education, BMI, sedentary behaviors, diabetes, hypertension, sleep duration into consideration (OR = 2.8, 95% CI: (1.4, 5.6) *p* = 0.004; Table 4). Finally, a non-liner relationship between log-transformed sNfL pg/mL and PHQ-9 scores were discovered with a log-likehood ratio test less than 0.05 (A. Figure 1 and A. Table 1). The results demonstrated that individuals with high levels of sNfL had a greater risk of depression symptoms, which may be associated with neuronal functional pathways being inhibited or tricked after suffering neuroaxonal damage. However, a significant proportion of participants with high incomes had greater depressive episodes, which is contrary to previous research.

No clear mechanism underlies the biological processes for the sNfL levels in the pathogenesis of neurodegenerative diseases; however, the existing evidence may provide powerful clues to its mechanism. There is a broad agreement that the measurement of plasma or serum levels of NfL in neurological disorders or neurodegenerative diseases act as indirect indicator of the degree of axonal injury [37]. In addition, NfL levels can affect the hippocampal morphology in animals, thereby contribute to depression [38, 39]. Those potential mechanisms contribute to our understanding of the role of NfL in identifying new therapies for depression.

Our study has several strengths. This report provides public health researchers with a comprehensive population-based assessment of sNfL concentrations in the U.S. population. In addition, the data collection and survey procedures for NHANES followed standardized protocols and were rigorously inspected for quality assurance. Notably, the correlation between sNfL and depression remains statistically significant even after considering multiple confounding factors, revealing a novel discovery presented here for the first time. Additionally, the sNfL levels underwent a logarithmic transformation to attain a normal distribution. Moreover, the assessment for depression was conducted using the PHQ-9, which has been identified as an objective and sensitive screening tool [1].

Several limitations should also be acknowledged. Firstly, despite controlling for confounding factors through various methods, such as multivariable regression, we were unable to eliminate the possibility of residual and unmeasured confounding, including cognitive functions and physical activity as sample size limitations. Moreover, the ongoing trials will provide further insights into the correlation between alterations in NfL levels and depression. Besides, although the PHQ-9 has been identified as an effective tool for screening depression, subjective factors among participants may have some influence. We would perfect our results in conjunction with other approaches in the future. In addition, MRI-based clinical studies have shown a correlation between disease activity and elevated levels of NfL, such as MS [40]. However, the efficiency of NfL in monitoring depression progression is not yet defined and we hope to take more imaging features into consideration in the future. Besides, due to the nature of cross-section, causality cannot be implied.

## Conclusion

Above all, in this large, population-based cross-sectional research, we provide evidence that sNfL was elevated in depression patients. Identifying the qualities of a delicate and minimally intrusive biomarker, such as neurofilament light protein, holds potential not only as a diagnostic biomarker for treatment-resistant major depression but also for monitoring disease progression and treatment effectiveness.

### Electronic supplementary material

Below is the link to the electronic supplementary material.

Supplementary Material 1

Supplementary Material 2

## Data Availability

The datasets generated and/or analyzed during the current study are available in the NHANES database (https://www.cdc.gov/nchs/nhanes/index.htm).
