# Supplementary material for: Serum neurofilament light chain levels are associated with depression among US adults: a cross-sectional analysis among US adults, 2013–2014
Source: BMC Psychiatry. 2024 Jul 24;24:527. doi: 10.1186/s12888-024-05964-0 (PMC11267666; doi:10.1186/s12888-024-05964-0)
Supplement: Supplementary file 1 — Supplementary Material 1 [file 12888_2024_5964_MOESM1_ESM.docx]

**A. Figure 2.** The association between log-transformed sNfL levels (pg/mL) and PHQ-9 scores.


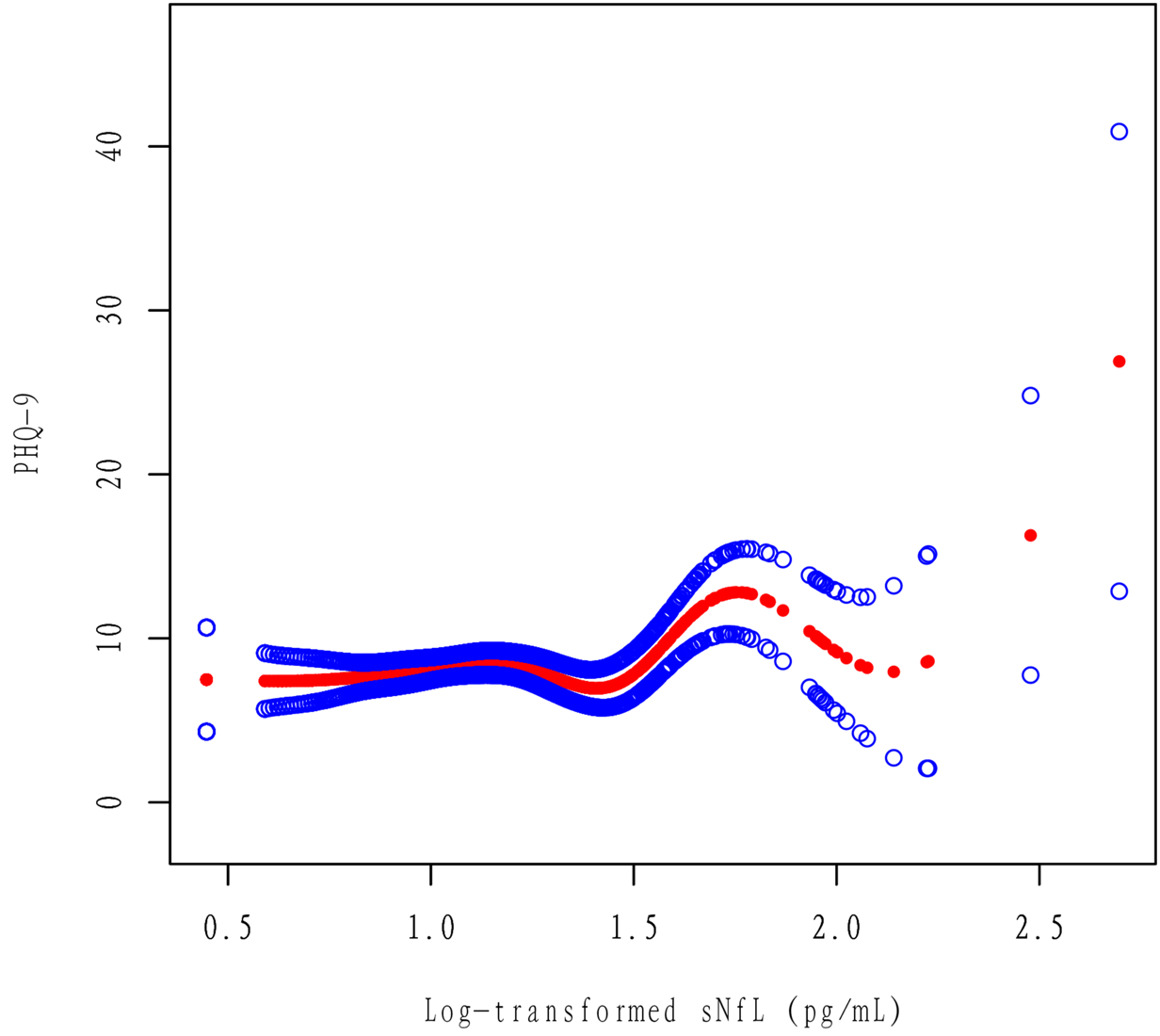


The solid red line represents the smooth curve fit between variables. Blue bands represent the 95% CI from the fit. All covariates were adjusted.
