# Supplementary material for: Serum neurofilament light chain levels are associated with depression among US adults: a cross-sectional analysis among US adults, 2013–2014
Source: BMC Psychiatry. 2024 Jul 24;24:527. doi: 10.1186/s12888-024-05964-0 (PMC11267666; doi:10.1186/s12888-024-05964-0)
Supplement: Supplementary file 2 — Supplementary Material 2 [file 12888_2024_5964_MOESM2_ESM.docx]

**A. Table 3.** Threshold effect analysis of log-transformed sNfL (pg/mL) on PHQ-9 scores.

| **PHQ-9 scores** | **β** | **(95%CI)** | **P-value** |
| --- | --- | --- | --- |
| **Model I** |  |  |  |
| Linear effect model | 1.9 | (0.1, 3.8) | 0.039 |
| **Model II** |  |  |  |
| <1.5 pg/mL | 0.3 | (-2.0, 2.5) | 0.807 |
| ≥1.5 pg/mL | 8.9 | (3.1, 14.7) | 0.003 |
| Log-likehood ratio test (LR Test) |  |  | 0.012 |

**Model I**: Linear effects model; **Model II**: Non-linear effects model.
